# Fluoxetine inhibited RANKL-induced osteoclastic differentiation *in vitro*

**DOI:** 10.1515/med-2024-1094

**Published:** 2024-12-17

**Authors:** Jing-wen Zhang, Fang-bing Zhao, Bing’er Ma, Xiao-qing Shen, Yuan-ming Geng

**Affiliations:** Department of Stomatology, Zhujiang Hospital, Southern Medical University, Guangzhou, China; East China Institute of Digital Medical Engineering, Shangrao, China

**Keywords:** osteoclast, receptor activator of nuclear factor kappa-B ligand, selective serotonin reuptake inhibitor, fluoxetine, orthodontic tooth movement

## Abstract

Selective serotonin reuptake inhibitor correlates with decreased bone mineral density and impedes orthodontic tooth movement. The present study aimed to examine the effects of fluoxetine on osteoclast differentiation and function. Human peripheral blood mononuclear cells (hPBMCs) and murine RAW264.7 cells were cultured with RANKL to stimulate osteoclast differentiation. The resulting multinucleated cells displayed characteristics of mature osteoclasts. Fluoxetine at 0.01–1 μM did not impact cellular viability or oxidative stress. However, 10 μM fluoxetine significantly reduced clonal growth, cell viability, and increased cytotoxicity and lipid peroxidation in RAW 264.7 cells. Further, application of 0.1 μM fluoxetine potently suppressed osteoclast differentiation of both RAW264.7 and hPBMCs, with reduced osteoclast numbers and downregulation of osteoclastic genes matrix metalloproteinase-9, cathepsin K, and integrin β3 at mRNA and protein levels. Fluoxetine also disrupted F-actin ring formation essential for osteoclast resorptive function. Mechanistically, fluoxetine inhibited NF-kB signaling by reducing phosphorylation of pathway members IκBα and p65, preventing IκBα degradation and blocking p65 nuclear translocation. In conclusion, this study demonstrates fluoxetine suppressing osteoclast differentiation in association with disrupting NF-kB activation, providing insight into orthodontic treatment planning for patients taking fluoxetine.

## 1 Introduction

Major depressive disorder (MDD) is a highly prevalent psychiatric illness that affects approximately 12% of the global population over their lifetime [1]. Selective serotonin reuptake inhibitors (SSRIs) have become first-line pharmacological interventions for MDD due to their clinical efficacy and favorable side effect profile [2]. By impeding the reuptake of serotonin in neuronal synapses, SSRIs increase extracellular serotonin levels, thus ameliorating depressive symptoms.

However, emerging clinical evidence indicates that extended SSRI usage correlates with decreased bone mineral density and heightened fracture susceptibility [3]. Additionally, animal studies demonstrate that SSRIs can impede orthodontic tooth movement (OTM) requiring mechanical forces [4]. While the mechanisms underlying these effects remain incompletely characterized, they likely involve SSRI-mediated serotonin signaling alterations that disrupt osteogenic and osteoclastic activity through serotonin receptors expressed in these bone-remodeling cell types [5].

Osteoclast-mediated bone resorption is critical in enabling OTM. Orthodontic forces lead to local inflammation and cytokine release, triggering osteoclast precursor recruitment and differentiation [6]. The receptor activator of nuclear factor kappa-B (RANK) ligand (RANKL) signaling pathway plays an essential role in osteoclast formation and activity. RANKL stimulation of the RANK receptor initiates downstream signaling cascades culminating in osteoclastogenesis [7]. In fact, studies show that inhibiting RANKL signaling directly reduces OTM rates in animal models [8].

Intriguing evidence suggests that SSRIs may interfere with RANKL pathway signaling. The serotonin-enhancing agent cisapride was shown to suppress RANKL-induced osteoclastogenesis and function in bone marrow-derived macrophages [9]. Additionally, a mouse study demonstrated that gut-derived serotonin triggered by a depression state enhances cancer cell bone metastasis through increased osteoclast activity via the RUNX2/PTHrP/RANKL pathway [10]. These collective findings imply that SSRI-mediated alterations can disrupt normal and pathological RANKL signaling governing osteoclast biology.

Therefore, this study aimed to explore the potential modulatory effects exerted by SSRIs on the RANKL pathway, to provide insights into safeguarding bone health during clinical SSRI usage.

## 2 Materials and methods

### 2.1 Cell culture

Human peripheral blood mononuclear cells (hPBMCs) were isolated from one of the co-authors (J.Z.) who had no recent infections or medications. Blood (10 mL) was drawn, anticoagulated with heparin, and processed within 4 h. Blood was diluted 1:1 with phosphate-buffered solution (PBS), layered onto Ficoll-Paque, and centrifuged at 2,000 rpm for 20 min. The mononuclear cell layer was collected, washed with PBS, and centrifuged at 1,000 rpm for 10 min, and the supernatant was discarded. The wash was repeated, cells were resuspended and counted, and 1 × 10^5^ cells were plated per well for morphology examination.

The murine monocyte/macrophage cell line RAW264.7 was purchased from The Cell Bank of Type Culture Collection of the Chinese Academy of Sciences.

Cells were cultured in RPMI 1640 media (Gibco) containing 10% fetal bovine serum (Gibco), 2 mM l-glutamine, 100 IU/mL penicillin, and 100 μg/mL streptomycin. Osteoclastogenesis was induced with 100 ng/mL sRANKL and 50 ng/mL M-CSF (both from PeproTech). Half of the medium was changed every 3–4 days.

### 2.2 Plate cloning assay

Cells were seeded at a density of 100 cells per well in a 6-well plate and cultured in groups for 10 days. They were then fixed with 4% paraformaldehyde for 15 min and stained with crystal violet solution for 10–30 min. After air-drying at room temperature, photographs were taken. The number of cell colonies was calculated using Image-Pro Plus 6.0 software (Media Cybernetics).

### 2.3 Cell counting kit-8 (CCK-8) assay

Cells were seeded at a density of 5 × 10^3^ cells per well in a 96-well plate and cultured for 2 days in groups. CCK-8 solution (Dojindo) was then added to each well, and the plates were incubated in the dark for 2 h. The optical density (OD) at 450 nm was measured for each well. Cell viability was calculated using the formula: [(experimental well OD – blank well OD)/(control well OD – blank well OD)] × 100%.

### 2.4 Lactate dehydrogenase (LDH) assay

Cells were plated at a density of 5 × 10^3^ cells per well in a 96-well plate and cultured for 2 days in designated groups. Following incubation, the plates were centrifuged at 400 × *g* for 5 min at room temperature to collect the supernatant. The supernatant was then transferred to a new 96-well plate. LDH activity was measured using an LDH assay kit (Beyotime), with the optical density (OD) recorded at a wavelength of 490 nm.

### 2.5 Malondialdehyde (MDA) assay

Cells were seeded at a density of 1 × 10^5^ cells per well in a 6-well plate and cultured in groups for 2 days, followed by protein extraction. Protein concentration was determined using a BCA assay kit (Beyotime). The MDA content was measured using an MDA assay kit (Beyotime) at an absorbance of 532 nm.

### 2.6 Tartrate-resistant acid phosphatase (TRAP) staining

Cells were seeded at 1 × 10^4^ cells per well on coverslips in a 24-well plate. Cells were induced osteoclast differentiation and cultured for 5 days. TRAP staining was performed using a TRAP staining kit (Bestbio). The average of TRAP-positive cell counts from six random fields of view is utilized to analyze the level of osteoclast differentiation.

### 2.7 Real-time quantitative polymerase chain reaction (qPCR)

Cells were washed with PBS and RNA extracted using TRIzol (Invitrogen), with purity confirmed by UV spectrophotometry. cDNA was synthesized with M-MLV kit (Promega) and qPCR was performed on an Applied Biosystems machine (Thermo Fisher Scientific) using ChamQ™ SYBR^®^ Master Mix (Vazyme Biotech). The 40-cycle protocol included 95°C for denaturation and 60°C for annealing/extension. GADPH was the reference gene, and mRNA levels were quantified using the 2^−ΔΔCq^ method. Primer details are in Table 1.

**Table 1:** Primer sequences used for quantitative real time polymerase chain reaction analysis

Mouse GAPDH	Forward	AAGAAGGTGGTGAAGCAGG
	Reverse	GAAGGTGGAAGAGTGGGAGT
Mouse MMP-9	Forward	GCAGAGGCATACTTGTACCG
	Reverse	TGATGTTATGATGGTCCCACTTG
Mouse CTSK	Forward	GAAGAAGACTCACCAGAAGCAG
	Reverse	TCCAGGTTATGGGCAGAGATT
Mouse integrin β3	Forward	CCACACGAGGCGTGAACTC
	Reverse	CTTCAGGTTACATCGGGGTGA
Human GAPDH	Forward	GCACCGTCAAGGCTGAGAAC
	Reverse	TGGTGAAGACGCCAGTGGA
Human MMP-9	Forward	GGGACGCAGACATCGTCATC
	Reverse	TCGTCATCGTCGAAATGGGC
Human CTSK	Forward	ACACCCACTGGGAGCTATG
	Reverse	GACAGGGGTACTTTGAGTCCA
Human integrin β3	Forward	GTGACCTGAAGGAGAATCTGC
	Reverse	CCGGAGTGCAATCCTCTGG

### 2.8 Western blot analysis

Cells were lysed in Tris-HCl buffer with 150 mM NaCl, 1 mM ethelene diamine tetra acetic acid, 1% Triton X-100, and protease inhibitors, following PBS washes. Protein concentrations were measured using a BCA assay kit (Beyotime). Proteins (20 μg) underwent 8% sodium dodecyl sulfate polyacrylamide gel electrophoresis and were transferred to polyvinylidene fluoride membranes, which were blocked with milk and 0.1% Tween-20. Membranes were probed with primary antibodies (anti-matrix metalloproteinase-9 [MMP-9] antibody, integrin β3 antibody, and cathepsin K antibody from Abcam; anti-IκB-α antibody, P65 antibody, and p-P65 antibody from CST) and then with goat anti-rabbit secondary antibody (Thermo Fisher). Bands were visualized using ECL (Forevergen) according to the manufacturer’s instruction. Anti-GAPDH antibody was used as the loading control (CST).

### 2.9 Immunofluorescence assay

Following cell culture and osteoclastogenesis induction, cells were allowed to adhere to confocal dishes and washed with PBS. They were then fixed with 4% paraformaldehyde, permeabilized with 0.2% Triton X-100, and blocked with 2% bovine serum albumin to prevent non-specific binding. Cells were incubated with Alexa Fluor^®^ 647 Anti-NF-kB p65 antibody (Abcam) at room temperature overnight. F-actin was stained with FTIC-labelled phalloidin (Beyotime) for 20 min at 37°C. Nuclei were stained with DAPI (Beyotime) for 5 min. After the final washes, cells were sealed with an anti-fade agent and visualized for P65 and F-actin localization using a microscope (Zeiss Imager Z1). The relative integrated intensity was calculated as the ratio of the integrated fluorescence intensity of P65 in the nuclear area to that of the total cell [11]. All cells in three random fields were analyzed using Image‑Pro Plus 6.0. F-actin rings were counted in six randomly selected fields of view, and the average count was used to assess the level of osteoclast differentiation.

### 2.10 Statistical analysis

Data were analyzed using IBM SPSS Statistics software, version 22.0. Quantitative data were expressed as mean ± standard deviation. One-way analysis of variance was employed, with an initial test for homogeneity of variances. Subsequent pairwise comparisons between groups were conducted; if variances were equal, the least significant difference (LSD) test was used, while in the case of unequal variances, the Welch correction followed by Dunnett’s T3 test was applied. A *p*-value of less than 0.05 was considered statistically significant.


**Informed consent:** Informed consent has been obtained from all individuals included in this study.
**Ethical approval:** The research related to human use has been complied with all the relevant national regulations, and institutional policies and in accordance with the tenets of the Helsinki Declaration, and has obtained approval from the Ethics Committee of Zhujiang Hospital, Southern Medical University (2024-KY-143-03).

## 3 Results

### 3.1 Multinucleated cell formation

**Figure 1:**
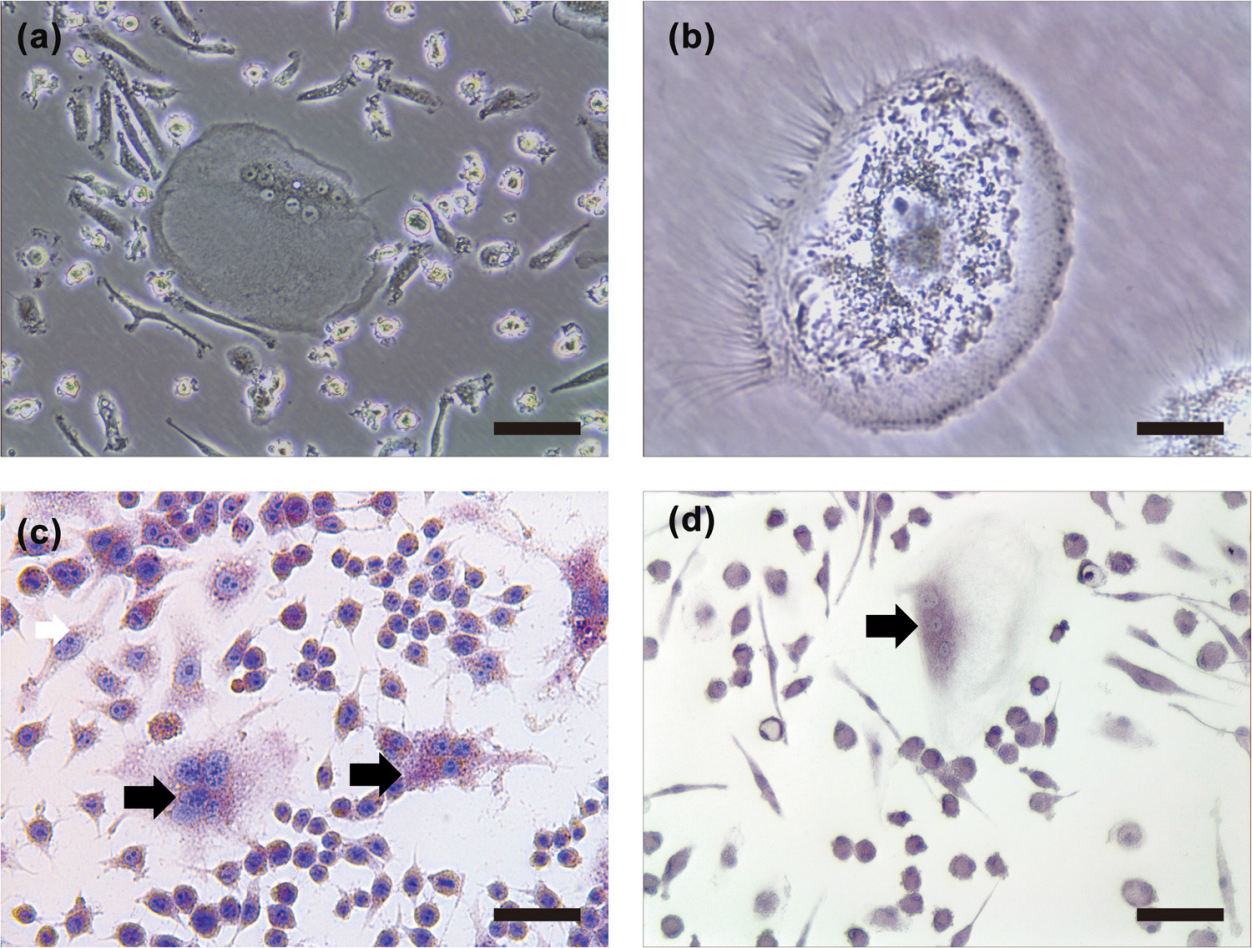
Multinucleated cell formation. (a) After RANKL-induced osteoclast differentiation, hPBMCs displayed distinct morphological changes. Scale bar, 50 μm. (b) Increased cell volume, irregular shape, multiple fused nuclei, and cell protrusions could be observed. Scale bar, 10 μm. (c) TRAP staining further revealed the presence of red-purple TRAP-positive granules in the cytoplasm and blue-stained nuclei within the osteoclast-like cells derived from hPBMCs. Scale bar, 50 μm. (d) Similar morphological features and TRAP staining patterns are also evident in RANKL-differentiated RAW264.7 murine macrophage cells. Scale bar, 50 μm. The arrows indicate TRAP-positive cells.

Primary hPBMCs were cultured with RANKL and M-CSF for 10 days to induce osteoclast differentiation. This process led to the formation of multinucleated cells characterized by increased size, cytoplasmic granularity, villous-like protrusions, and irregular shapes, all of which contained multiple nuclei (Figure 1a and b), as confirmed by TRAP staining (Figure 1c). Under the same osteoclastogenic conditions, the RAW264.7 murine monocytic cell line also developed TRAP + multinucleated osteoclasts (Figure 1d).

### 3.2 Fluoxetine’s concentration-dependent effects

**Figure 2:**
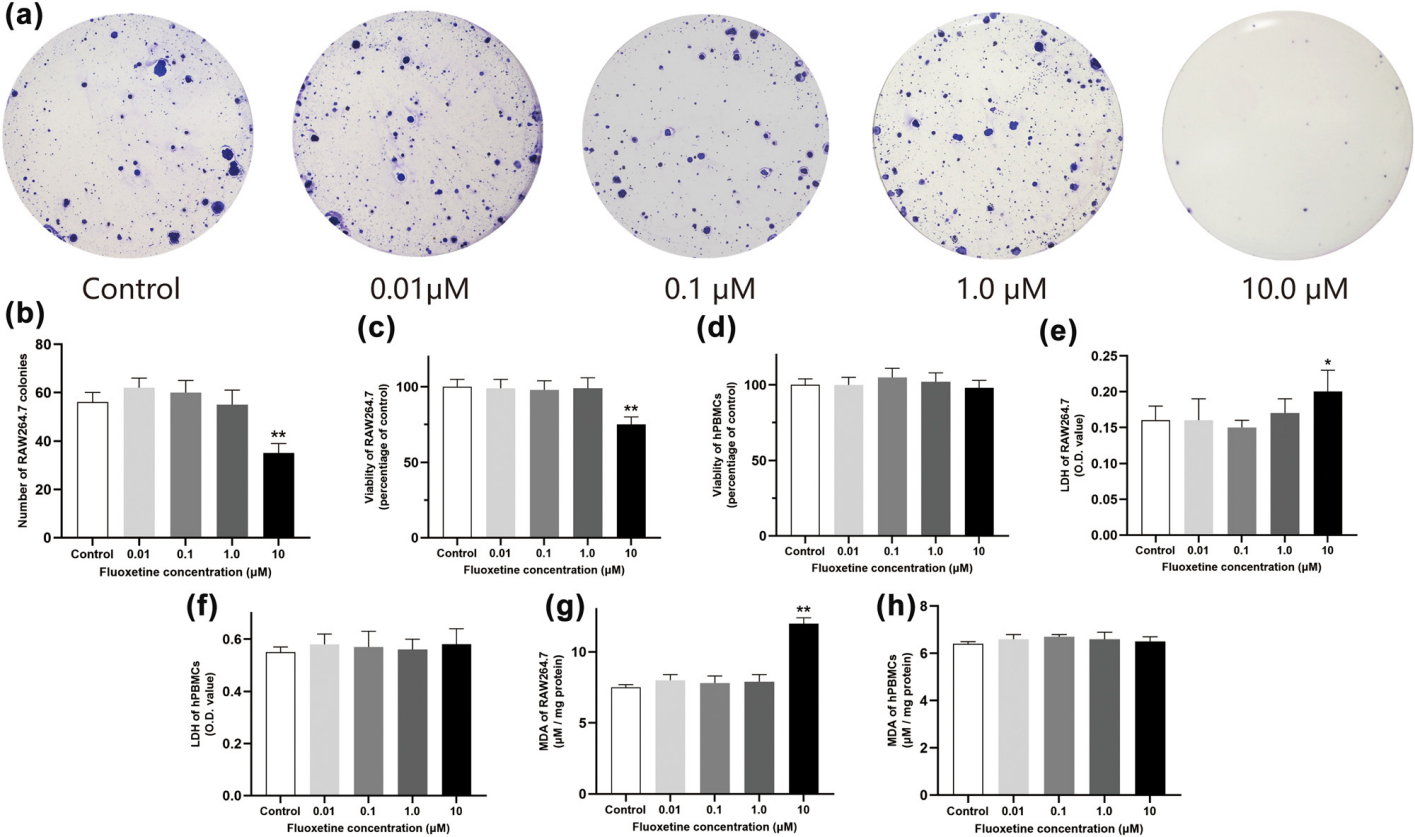
Fluoxetine’s concentration-dependent effects. (a) Adding 0.01–1 μM fluoxetine to RAW264.7 for 10 days did not affect the number of cell clones versus control. (b) The 10 μM group had significantly decreased clones of RAW264.7 (*n* = 6, *P* < 0.001). (c) After 10 days, 10 μM fluoxetine significantly reduced RAW264.7 viability by CCK-8 assay (*n* = 3, *P* = 0.0015). (d) Fluoxetine did not affect hPBMC viability (*n* = 3, *P* = 0.5739). (e and g) The 10 μM fluoxetine RAW264.7 group had significantly elevated LDH release and MDA levels versus other groups by LDH and MDA assays (*n* = 3; *P* = 0.0274 and <0.001, respectively). (f and h) Fluoxetine did not significantly affect LDH or MDA in hPBMCs (*n* = 3; *P* = 0.8108 and 0.1872, respectively). *P < 0.05, **P < 0.01.

Previous studies have not provided conclusive evidence regarding the effective concentration range of fluoxetine on osteoclasts. Moreover, the therapeutic dosage of fluoxetine in humans cannot be directly extrapolated to determine the appropriate concentration for *in vitro* cellular experiments. To explore fluoxetine’s concentration-dependent effects, 0.01–10 μM fluoxetine was added to the culture medium. In RAW264.7 cells, 10 μM fluoxetine significantly reduced clonal growth (Figure 2a and b), decreased viability in the CCK-8 test (Figure 2c), and increased cytotoxicity as shown in the LDH assay (Figure 2e) and lipid peroxidation in the MDA assay (Figure 2g). In contrast, hPBMCs showed no alterations in viability (Figure 2d), cytotoxicity (Figure 2f), or lipid peroxidation (Figure 2h) across the same concentration range.

### 3.3 Fluoxetine inhibits osteoclast differentiation

**Figure 3:**
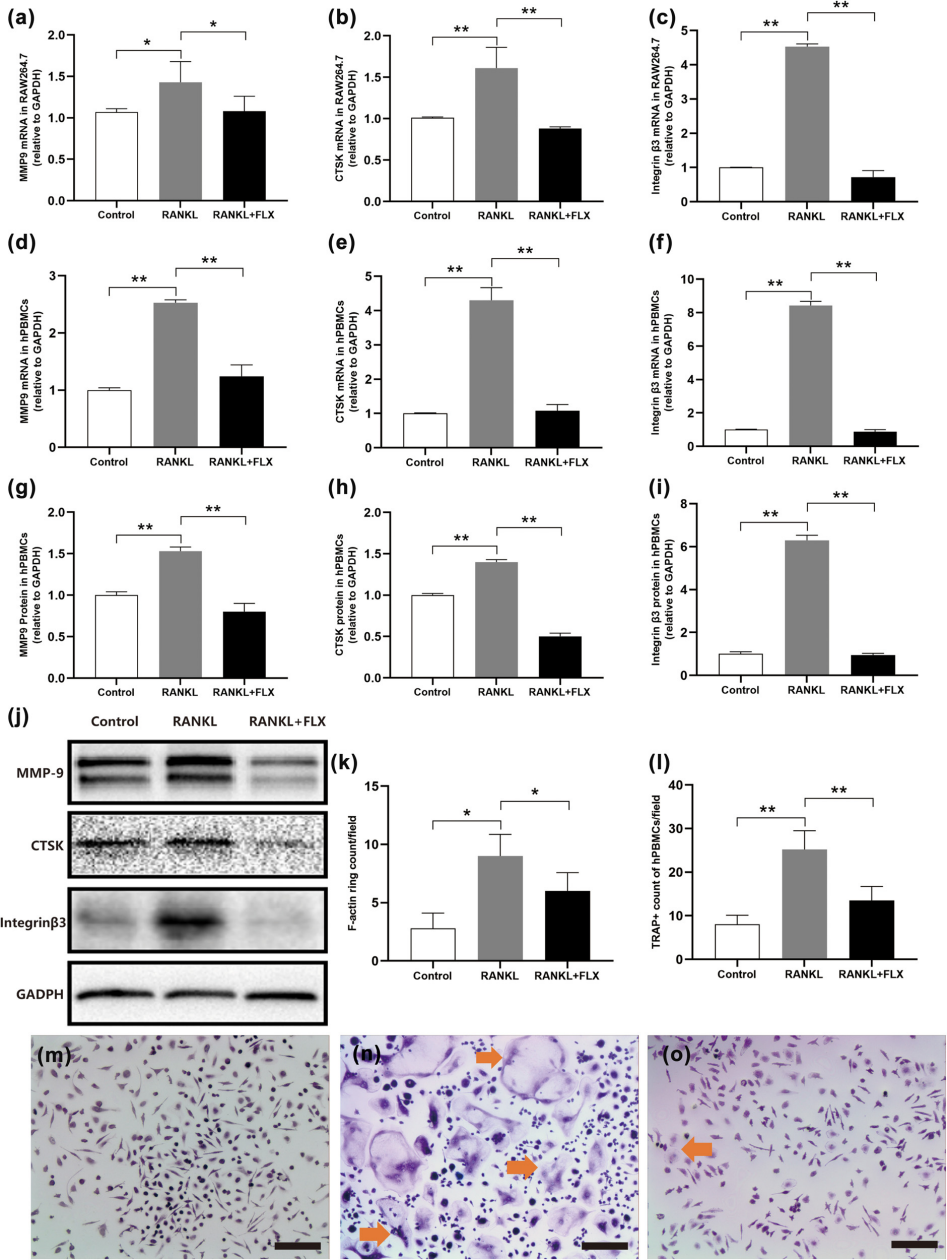
Fluoxetine inhibits osteoclast differentiation. After RANKL induction of RAW264.7, PCR detection showed upregulated mRNA expression of MMP-9 (a), CTSK (b), Integrin β3 (c), and fluoxetine treatment downregulated the gene expression levels (*n* = 3; *P* = 0.0151, 0.0018 and <0.001, respectively). In hPBMCs, RANKL also upregulated MMP-9 (d), CTSK (e), and Integrin β3 (f) mRNA expression, which were downregulated after fluoxetine treatment (*n* = 3; *P* < 0.001). Immunoblotting showed upregulated protein expression of CTSK, Integrin β3, and MMP9 in RANKL-induced hPBMCs, which were significantly downregulated after fluoxetine treatment (j), with statistically significant differences by semi-quantitative analysis (g–i) (*n* = 3; *P* < 0.001). The F-actin ring formation increased in RANKL-induced hPBMCs and was significantly reduced by fluoxetine treatment (k) (*n* = 6; *P* = 0.0012). The presentative images are shown in Figure 4. TRAP staining positive cells increased after RANKL induction (n) but were decreased in number after fluoxetine treatment (o), in comparison with the control (m). The difference among groups was significant statistically (l) (*n* = 6; *P* = 0.008). Scale bar, 50 μm. *P < 0.05, **P < 0.01.

**Figure 4:**
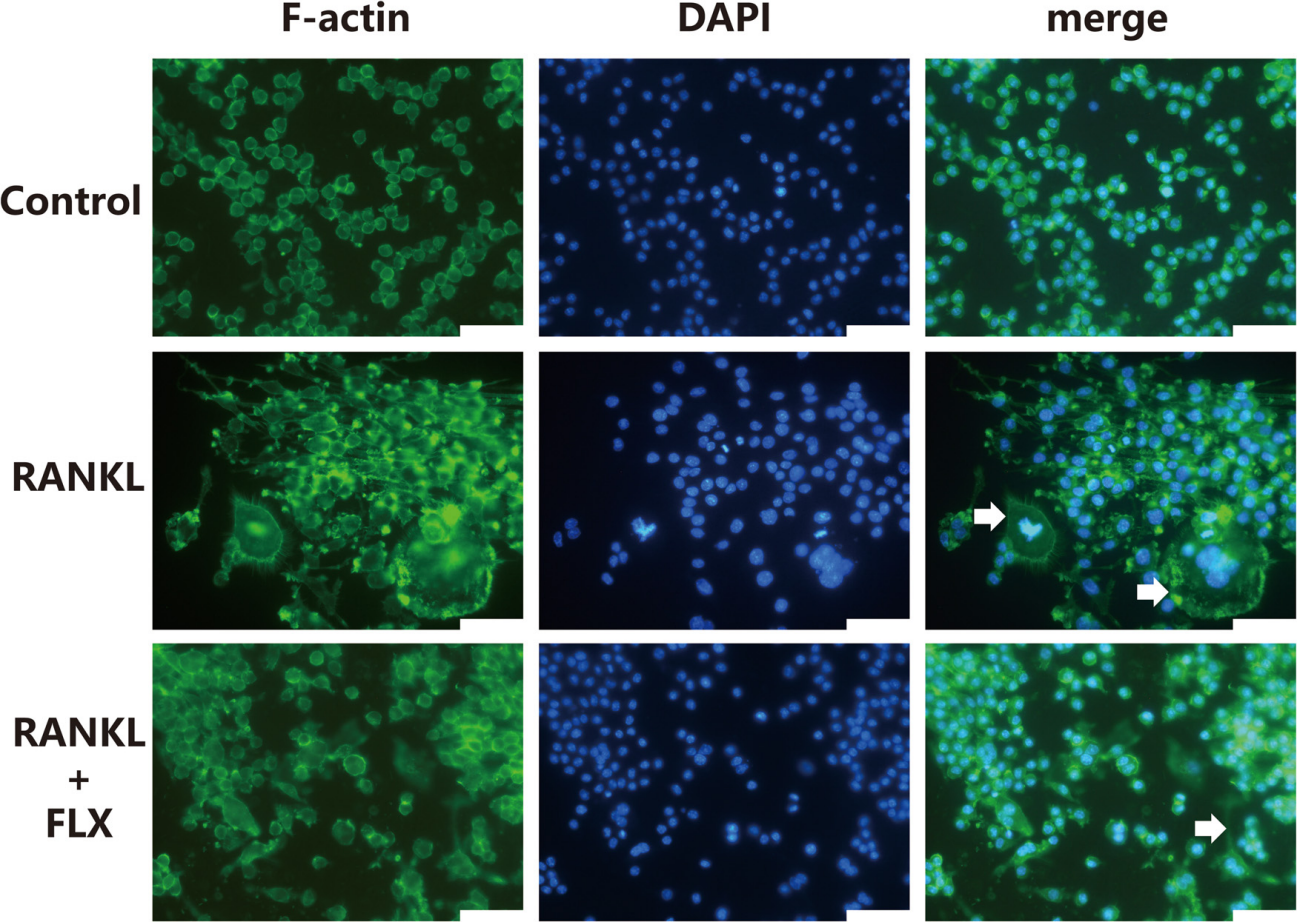
Immunofluorescence observation of F-actin. The arrows indicate the F-actin rings. Scale bar, 50 μm.

Based on its concentration-dependent effects, a fluoxetine concentration of 0.1 μM was chosen for subsequent experiments. RANKL induced osteoclast-specific genes such as MMP-9, cathepsin K, and integrin β3 at the mRNA level in both RAW264.7 cells (Figure 3a–c) and primary hPBMCs (Figure 3d–f). However, the introduction of fluoxetine markedly decreased the expression of these genes. Corresponding protein levels of MMP-9, cathepsin K, and integrin β3 showed similar reductions in hPBMCs as evidenced by immunoblotting (Figure 3g–j). TRAP + multinucleated osteoclasts from hPBMCs were seen upon RANKL and M-CSF culture, with a noticeable reduction in the presence of fluoxetine (Figure 3k–n). Furthermore, fluoxetine disrupted the formation of F-actin rings necessary for osteoclast function, visualized using immunofluorescence microscopy (Figure 4). These findings suggest that fluoxetine’s inhibition of osteoclast differentiation is closely linked to its impact on cellular structures and gene expression.

### 3.4 Fluoxetine disrupts NF-kB signaling

**Figure 5:**
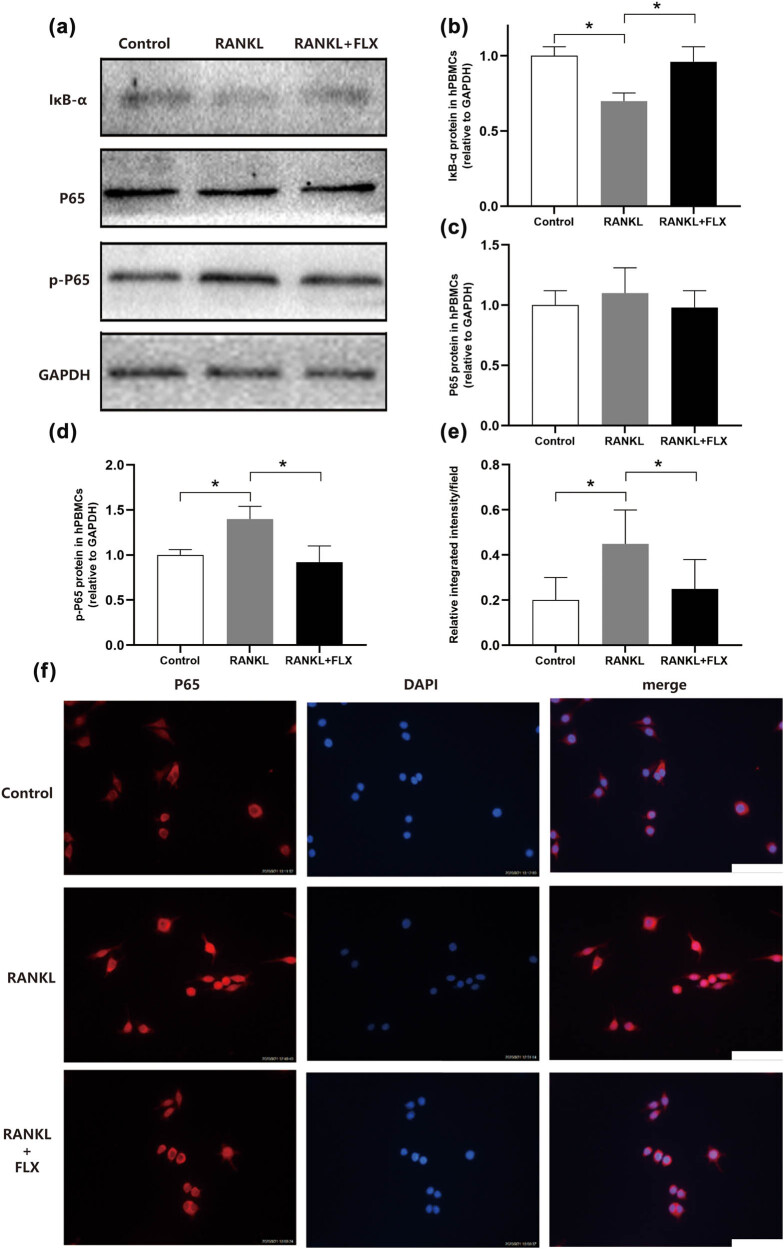
Fluoxetine disrupts NF-kB signaling. NF-kB signaling proteins were assayed with immunoblotting (a). After RANKL induction, IκB-α expression was downregulated and increased in response to fluoxetine treatment (b) (*n* = 3, *P* = 0.005). P65 expression was slightly upregulated after RANKL induction but without significant changes among groups (c) (*n* = 3, *P* = 0.6427). Phosphorylated P65 expression significantly increased after RANKL induction and decreased after fluoxetine treatment (d) (*n* = 3, *P* = 0.0105). Immunofluorescence observation of P65 intracellular localization in hPBMCs showed that compared to blank control, RANKL induction significantly enhanced red fluorescence of nuclear P65; red fluorescence intensity in the nucleus was significantly weakened after fluoxetine treatment (f). The difference among groups was significant statistically (e) (*n* = 6, *P* = 0.0099). Scale bar, 50 μm. *P < 0.05, **P < 0.01.

The addition of RANKL resulted in notable downregulation of IκB-α expression, which was subsequently restored with fluoxetine treatment (Figure 5b). Although P65 expression saw a slight increase following RANKL induction, this did not reach statistical significance (Figure 5c). Phosphorylated P65 expression was significantly increased after RANKL induction but was decreased by fluoxetine treatment (Figure 5d). Immunofluorescence analysis revealed that RANKL greatly enhanced the nuclear localization of P65 in hPBMCs, indicated by increased red fluorescence compared to control, while this intensity was significantly reduced after fluoxetine treatment (Figure 5e). The disruption of NF-kB signaling by fluoxetine provides a molecular explanation for its inhibitory effects on osteoclast differentiation observed in Section 3, highlighting a potential mechanism through which fluoxetine modulates osteoclastogenesis.

## 4 Discussion

This study presents new evidence that the commonly prescribed SSRI, fluoxetine, can directly hinder osteoclast differentiation at clinically relevant concentrations by disrupting NF-kB activation. By employing hPBMCs for their human physiological relevance and RAW264.7 cells for their established role in osteoclast research, we adopted a comprehensive approach to explore fluoxetine’s impact on osteoclast differentiation. Our findings demonstrate that fluoxetine inhibits the differentiation of primary human osteoclast precursors and murine RAW264.7 cells induced by RANKL, accompanied by reduced expression of genes and proteins essential for bone resorption. Furthermore, we show that fluoxetine attenuates multiple steps in the NF-kB signaling pathway, a crucial downstream mechanism activated by RANKL to promote osteoclastogenesis. Overall, this study highlights the intricate relationship between the serotonergic system and bone health, offering significant insights for patients undergoing SSRI therapy.

As an antidepressant, SSRIs often require long-term use. The steady-state plasma concentration of fluoxetine after 4 weeks of daily 20 mg administration is 0.540 ± 0.282 μM (https://www.nmpa.gov.cn/wwwroot/hy5/110.htm). Therefore, we examined the effects of 0.01–10 μM fluoxetine on cell viability. In line with clinical dosing, fluoxetine at 0.01–1 μM did not alter clonogenicity, cell viability, oxidative stress, or cytotoxicity. However, higher concentrations of 10 μM fluoxetine showed significant cytotoxicity in RAW264.7 cells. While highlighting the need for further research into differential cellular responses, the toxicity assessment suggested a safe concentration range for studying its effects on osteoclast differentiation without cytotoxic interference, in subsequent experiments, we selected a concentration of 0.1 μM fluoxetine with reasonable clinical relevance for further osteoclast interventions.

Selective serotonin reuptake inhibitors (SSRIs) have been shown to reduce bone mineral density and increase fracture risk with long-term use [12]. As several dental treatments rely on bone remodeling, the use of SSRIs is an important consideration for dentists [13]. Animal studies have demonstrated that SSRIs can alter the rate of OTM [14]. The underlying mechanism for this effect may be related to the ability of SSRIs to suppress osteoclast activity [15]. Osteoclasts are cells responsible for bone resorption, which is a necessary process for mechanical force-induced tooth movement during orthodontic treatment. Our findings show that the SSRI fluoxetine can directly inhibit the expression of key osteoclast genes including TRAP, cathepsin K, MMP-9, and integrin β3.

TRAP is highly expressed in osteoclasts and used as a histochemical marker. It prompts the dephosphorylation of bone matrix phosphoproteins including osteopontin and bone sialoprotein [16]. Cathepsin K is a cysteine proteinase suggested to be responsible for the proteolytic activation of TRAP [17]. Integrin β3 is an osteoclast cell-surface receptor involved in actin ring formation [18]. MMP-9, secreted by osteoclasts, has an important role in degrading the extracellular matrix [19]. By suppressing these osteoclast activities, SSRIs may hamper the bone remodeling required for efficient OTM. As a result, orthodontic patients receiving SSRI therapy may require extended treatment durations. However, further translational studies in humans are necessary to fully examine the effects of SSRIs on OTM.

Osteoclast differentiation is regulated by multiple signaling pathways, with NF-κB signaling being critical. Our data showed that in RANKL-stimulated preosteoclasts, fluoxetine suppressed NF-κB activation, as evidenced by inhibited IκBα degradation and p65 nuclear translocation. This implies that fluoxetine may modulate osteoclastogenesis by regulating NF-κB signaling. Previous studies have reported that serotonin activates the NF-κB pathway [20]. Intriguingly, RAW264.7 cells lack enzymes for de novo serotonin synthesis, indicating the requirement for an exogenous serotonin source. Considering SSRIs act on serotonin transporter (SERT) to block serotonin reuptake rather than directly interacting with serotonin receptors, our data suggest that SERT may contribute to the inhibitory effect of fluoxetine on the NF-κB pathway. It has been found that the SERT was upregulated during RANKL-induced osteoclast differentiation, implying its potential role in modulating the NF-κB pathway [21]. However, the exact mechanisms whereby SERT regulates osteoclasts remain unclear and warrant further investigation.

Several limitations should be acknowledged when interpreting our mechanistic *in vitro* findings. The study exclusively utilized an *in vitro* model of osteoclastogenesis, which cannot fully recapitulate complex *in vivo* skeletal physiology. More importantly, we only examined the effects of fluoxetine in isolation; SSRIs are often prescribed alongside other psychotropics like serotonin-norepinephrine reuptake inhibitors, warranting analysis of drug-drug interactions.

## 5 Conclusion

This *in vitro* study shows that SSRI fluoxetine impairs osteoclast differentiation by suppressing NF-kB signaling and osteoclast gene expression critical for bone resorption. We demonstrate fluoxetine attenuates multiple steps in the RANKL-induced NF-kB cascade during osteoclast formation. These findings offer insights into complex serotonin-bone interactions, with implications for skeletal health in long-term SSRI users regarding bone density and fracture risk.
